# Evaluation of the Applicability of an Overseas-Trained Artificial Intelligence System for Mammography Interpretation in Japan

**DOI:** 10.7759/cureus.101466

**Published:** 2026-01-13

**Authors:** Maya Makita, Kouzou Murakami, Wakana Murakami, Hiroko Takamatsu, Kanai Takahiro, Atsuhito Sekimoto, Yoshinori Ito, Yoshimitsu Ohgiya

**Affiliations:** 1 Department of Radiology, Showa Medical University, Tokyo, JPN; 2 Radiology, Nanahoshi Clinic, Chiba, JPN

**Keywords:** artificial intelligence, breast neoplasms, computer-aided detection, diagnostic imaging, external validation, japan, mammography, reader study

## Abstract

Background: Breast cancer remains a major public health issue in Japan, and artificial intelligence (AI)-based computer-aided detection (CAD) systems have the potential to enhance diagnostic performance. We conducted a two-phase evaluation of an AI-CAD trained on non-Japanese data: an external validation using Japanese mammography images and a reader study assessing its impact on diagnostic performance.

Methods: We performed an external validation to evaluate the diagnostic performance of a commercial AI-CAD system using full-field digital mammography (FFDM) images obtained from Japanese patients. This study primarily focused on evaluating the standalone diagnostic performance of an AI-CAD system using a validation cohort of 338 Japanese patients. To further assess its practical utility, a supplementary multi-reader study with 40 selected cases was conducted to observe the interaction between radiologists and AI output. The AI-CAD was developed and trained outside Japan. Diagnostic performance was assessed using sensitivity, specificity, and receiver operating characteristic curve analysis.

Results: On validation data, the AI-CAD achieved a sensitivity of 79%, specificity of 89%, and an area under the curve (AUC) of 0.897 (95% CI 0.860-0.934). In a reader study of 40 cases, their performance improved from an AUC of 0.750 to 0.756 (Breast Imaging Reporting and Data System (BI-RADS); p=0.505) and from 0.750 to 0.761 (Likelihood of Malignancy; p=0.110) when assisted by AI-CAD.

Conclusions: Although no statistically significant difference was observed, AI‐aided readings yielded AUCs comparable to AI-unaided readings (95% CI overlap); these findings suggest the feasibility of applying an AI‑CAD trained outside Japan to Japanese cases, while larger prospective screening studies are required to establish clinical impact.

## Introduction

Breast cancer remains a major cause of cancer-related mortality among women worldwide, although advances in early detection and treatment have substantially improved patient outcomes [1]. In parallel with these developments, artificial intelligence (AI)-based tools have been increasingly introduced to support the interpretation of digital mammography, aiming to enhance diagnostic accuracy [2-4]. Notably, the majority of commercially available AI systems have been trained predominantly on datasets collected outside Japan.

Although several commercial AI-based computer-aided detection (CAD) systems, such as those developed by HOLOGIC [5], iCAD [6], and CureMetrix [7], have received regulatory approval outside Japan, evidence regarding their clinical performance in Japanese populations remains limited. Although current clinical guidelines describe the potential role of AI-CAD in mammography interpretation, they also note that evidence derived from Japanese populations remains limited [8]. This underscores the need to evaluate AI-CAD performance directly on domestic digital mammography images to determine its true utility in routine practice [8]. The Lunit INSIGHT MMG (v1.1.7.2; Lunit, Seoul, South Korea) is an AI-based CAD system built on a deep convolutional neural network architecture and trained using a large-scale, multi-national dataset [9]. Detailed distributions of patient race and age in the training dataset are not publicly reported. Given the potential influence of racial and demographic differences on imaging findings, it is important to validate its applicability within Japanese populations. Accordingly, this study evaluated the breast cancer detection performance of the Lunit INSIGHT MMG using mammography images from Japanese women and examined whether AI assistance influences physicians’ diagnostic accuracy.

## Materials and methods

Digital mammography examinations included in this study were retrospectively identified from patients evaluated at Showa Medical University Hospital. Study approval was obtained from the Ethics Review Committee of Showa Medical University (Approval No. 3426), and an opt-out consent procedure was applied with public disclosure on the institutional website. Mammographic interpretation was performed using category classification and background breast density assessment in accordance with the Breast Imaging Reporting and Data System (BI-RADS) guidelines [10]. All study procedures complied with the Declaration of Helsinki and relevant local regulations. The study design and reporting adhered to Standards for Reporting of Diagnostic Accuracy Studies (STARD) 2015 and the Checklist for Artificial Intelligence in Medical Imaging (CLAIM) 2024 update; STARD‑AI is an in‑development protocol, and relevant items were considered where applicable [11-13].

**Figure 1:**
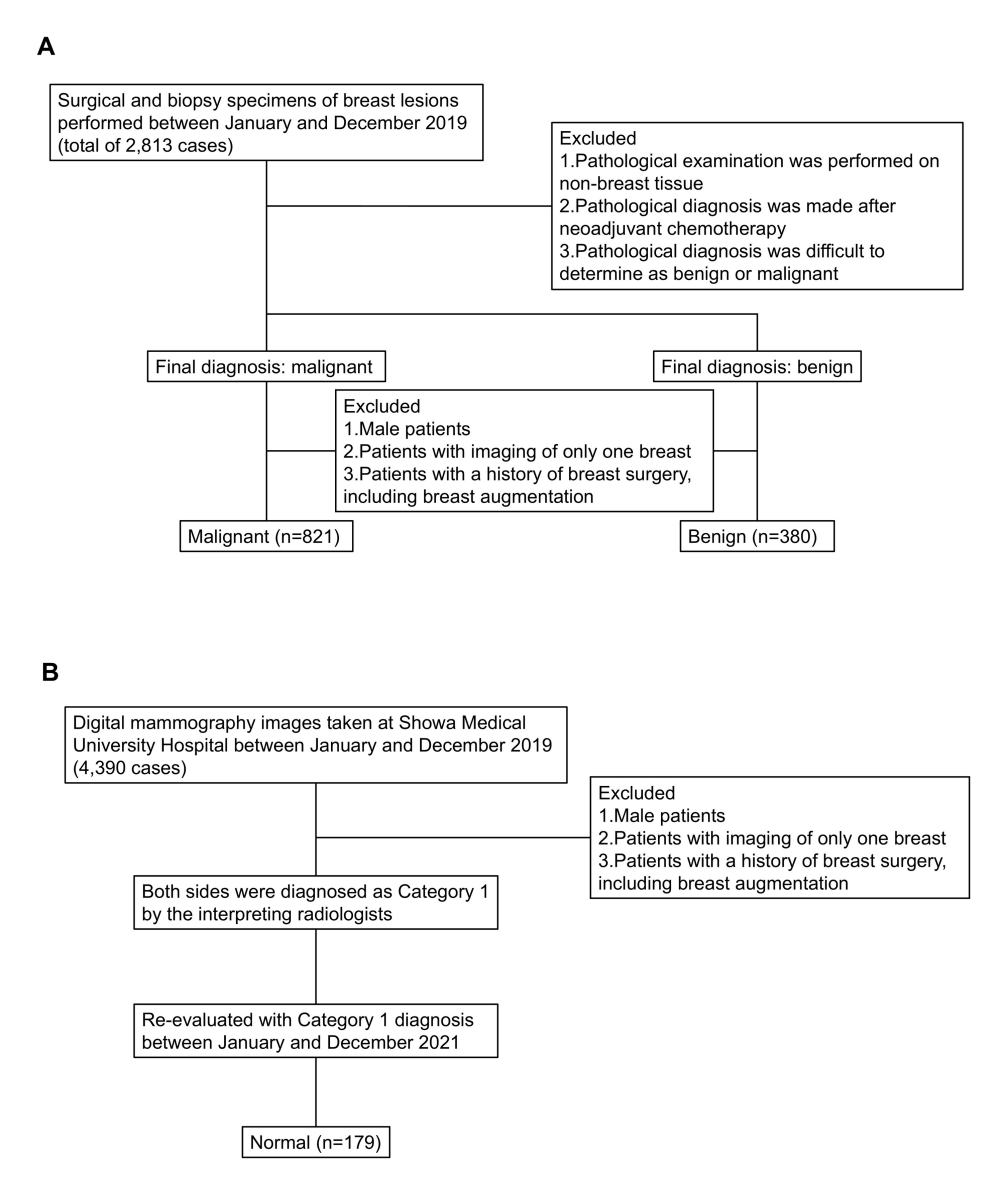
Case selection and group definitions. (A) Flow diagram depicting the process used to select malignant and benign cases based on surgical and biopsy specimens of breast lesions obtained between January and December 2019. Cases were excluded if pathological examination was performed on non-breast tissue, diagnosis was made after neoadjuvant chemotherapy, or pathological classification as benign or malignant was difficult. Additional exclusions included male patients, unilateral imaging only, and a history of breast surgery. (B) Flowchart illustrating the selection of normal cases from digital mammography examinations performed during the same period. Male patients, unilateral imaging, and prior breast surgery were excluded. Cases initially interpreted as BI-RADS category 1 were re-evaluated and confirmed as normal. BI-RADS, Breast Imaging Reporting and Data System [10].

The investigation consisted of two sequential components. Initially, an external validation was performed to assess the diagnostic performance of the AI-CAD system using digital mammography images from Japanese patients. Second, we conducted a sequential multi-reader, multi-case (MRMC) study in which the same 40 cases (21 malignant, nine benign, 10 normal; 80 breasts in total) were randomly selected from the validation dataset to evaluate how AI assistance affects diagnostic performance by human readers. In Japan, both radiologists and breast surgeons are commonly involved in interpreting mammographic screening studies. Therefore, we included both groups as participants in the reader study to reflect real-world clinical practice. The case selection process and group definitions are illustrated in Figure 1.

The AI-CAD system was applied to full-field digital mammography (FFDM) images. The AI-processed images were static and not zoomable. However, during the gold standard (GS) assignment phase, the radiologists had full access to the original FFDM images, which could be freely manipulated (e.g., zoom, contrast adjustment) to support accurate interpretation. In both steps, readers used the same Digital Imaging and Communications in Medicine (DICOM) viewer with full window/level and zoom. Step 1 (unaided) used original FFDM only (no AI overlays). Step 2 (AI-aided) overlaid the AI heatmaps and abnormality score on the same images; no other changes to the viewing environment were introduced. After this initial assessment, they were allowed to refer back to the original FFDM images in a toggled manner, enabling side-by-side evaluation. This setup aimed to simulate realistic reading conditions where radiologists might refer to unprocessed images even when AI suggestions are presented.

Computer-aided diagnosis software

The AI-CAD system evaluated in this study was Lunit INSIGHT MMG, which was developed using a convolutional neural network and trained on a large multinational dataset [4,9]. Previous studies have shown that it outperforms radiologists in detecting breast cancer from mammography images and significantly improves diagnostic accuracy when combined with AI assistance [4,9,14-18]. It detects regions suggestive of breast cancer on mammography images and marks areas indicative of malignant lesions. The system displays an abnormality score (range 0-100) for qualitative assessment, which assists radiologists in the diagnosis.

Patients and dataset

Two datasets were prepared for analysis. One dataset consisted of breast lesions that underwent surgical resection or biopsy between January and December 2019, comprising 2,813 cases, whereas the other dataset included 4,390 digital mammography examinations performed during the same period at Showa Medical University Hospital. Based on the reference standards applied to each dataset, cases were categorized as malignant, benign, or normal.

Malignant and benign cohorts consisted of diagnostic, pathology-proven cases, whereas the “normal” cohort was defined as BI-RADS 1 both at baseline and at two-year follow-up. Thus, the study dataset was intentionally enriched and does not reflect the prevalence of population-based screening.

From Group A, malignant and benign cohorts were defined based on final pathological diagnoses obtained from surgical or biopsy specimens.

From Group B, we defined the normal group as those whose bilateral mammography images were classified as BI-RADS category 1 in 2019 and who also had BI-RADS 1 findings in 2021. To ensure inclusion of unequivocally normal cases suitable for evaluating the standalone performance of the AI-CAD, we restricted the normal group to those consistently classified as BI-RADS 1. Cases assigned to BI-RADS categories 2 or 3 - potentially including lesions initially judged indeterminate but later confirmed benign - were excluded to eliminate interpretive ambiguity. Although the two readings were approximately two years apart, recall bias was minimized because the interpreting radiologists were not necessarily the same.

Cases were excluded if patients were male, had unilateral mammography only, or had a history of breast surgery, including augmentation. For malignant and benign cases, additional exclusions were applied when pathological assessment involved non-breast tissue, followed neoadjuvant chemotherapy, or did not allow confident differentiation between benign and malignant disease. Tumor staging was assigned according to the Union for International Cancer Control (UICC) TNM Classification (8th edition) [19]. After applying these criteria, 821 malignant, 380 benign, and 179 normal cases were initially identified. From each group, examinations were selected in chronological order, followed by further exclusions related to image analyzability or extended intervals between imaging and pathological diagnosis. The final dataset comprised 119 malignant, 95 benign, and 124 normal cases. From the verification data, we randomly selected 40 cases (21 malignant, nine benign, and 10 normal) for reader study. Details of the reader study protocol are described in the following section. Patient demographics and imaging characteristics for the validation cohort and the reader study cohort are summarized in Tables 1-6.

**Table 1:** Patient characteristics of the validation cohort Patient-level characteristics of the validation cohort (N = 338 patients). Values are presented as number (%) unless otherwise indicated. Breast density was assessed according to the Breast Imaging Reporting and Data System (BI-RADS) classification [10].

Characteristic	Overall N=338	Malignant n=119	Benign n=95	Normal n=124
Age group, n(%)				
≤50 years	159 (47%)	58 (49%)	71 (75%)	30 (24%)
51–65 years	112 (33%)	34 (29%)	19 (20%)	59 (48%)
>65 years	67 (20%)	27 (23%)	5 (5%)	35 (28%)
Breast Density grade, n(%)				
Fatty	4 (1%)	2 (2%)	1 (1%)	1 (1%)
Scattered fibroglandular dense	113 (33%)	55 (46%)	29 (31%)	29 (23%)
Heterogeneous dense	210 (62%)	54 (45%)	62 (65%)	94 (76%)
Extremely dense	11 (3%)	8 (7%)	3 (3%)	0 (0%)

**Table 2:** Imaging characteristics of breasts in the validation cohort Breast-level imaging characteristics of the validation cohort (N = 676 breasts). Radiologist interpretation was assessed according to the Breast Imaging Reporting and Data System (BI-RADS) classification [10]. The abnormality score was generated by the AI-CAD system (Lunit INSIGHT MMG) [4,9], as described in the Methods section. AI-CAD, artificial intelligence-based computer-aided diagnosis system; DCIS, ductal carcinoma in situ. ^a^ Challenging to determine as invasive or noninvasive.

Characteristic	Overall N=676	Cancer n=127	Not cancer n=549
Interpretation of the radiologists, n(%)			
Category 1, 2	507 (75%)	10 (8%)	497 (91%)
Category 3, 4, 5	169 (25%)	117 (92%)	52 (9%)
AI-CAD abnormality score, n(%)			
<10%	520 (77%)	27 (21%)	493 (90%)
≥10%	156 (23%)	100 (79%)	56 (10%)
Histology, n(%)			
Invasive cancers	—	102 (80%)	—
DCIS	—	22 (17%)	—
Papillary carcinoma (encapsulated)	—	1 (1%)	—
Special type	—	1 (1%)	—
Others ^a^	—	1 (1%)	—

**Table 3:** Mammographic findings in the validation cohort Mammographic findings are presented on a breast basis. Because multiple findings could be present in a single breast, the total number of findings does not correspond to the total number of breasts.

Mammography findings	Cancer	Not cancer
Mass, n	50	24
Asymmetry, n	34	13
Distortion, n	62	3
Calcifications, n	55	26

**Table 4:** Patient characteristics of the reader study cohort Patient-level characteristics of the reader study cohort (N = 40 patients). Values are presented as number (%) unless otherwise indicated. Breast density was assessed according to the Breast Imaging Reporting and Data System (BI-RADS) classification [10].

Characteristic	Overall N=40	Malignant n=21	Benign n=9	Normal n=10
Age group, n(%)				
≤50 years	20 (50%)	12 (57%)	6 (67%)	2 (20%)
51–65 years	16 (40%)	7 (33%)	2 (22%)	7 (70%)
>65 years	4 (10%)	2 (10%)	1 (11%)	1 (10%)
Breast Density grade, n(%)				
Fatty	0 (0%)	0 (0%)	0 (0%)	0 (0%)
Scattered fibroglandular dense	14 (35%)	7 (33%)	4 (44%)	3 (30%)
Heterogeneous dense	24 (60%)	12 (57%)	5 (56%)	7 (70%)
Extremely dense	2 (5%)	2 (10%)	0 (0%)	0 (0%)

**Table 5:** Breast-level imaging characteristics of cases included in the reader study Breast-level imaging characteristics of cases included in the reader study. ^a ^Tumor staging was determined according to the UICC TNM Classification of Malignant Tumours, 8th edition [19]. UICC, Union for International Cancer Control; TNM, tumour–node–metastasis; DCIS, ductal carcinoma in situ; T, primary tumor; N, regional lymph nodes; M, distant metastasis.

Characteristic	Cancer n=23
Histology, n(%)	
Invasive cancers	15 (65%)
DCIS	7 (30%)
Special type	1 (4%)
UICC T classifications^ a^, n(%)	
Tis(DCIS)	7 (30%)
T1mi	2 (9%)
T1a	2 (9%)
T1b	2 (9%)
T1c	2 (9%)
T2	6 (26%)
T3	0 (0%)
T4a	1 (4%)
unknown	1 (4%)
UICC N classifications^ a^, n(%)	
N0	19 (83%)
N1	3 (13%)
unknown	1 (4%)

**Table 6:** Mammographic findings in the reader study cohort Mammographic findings in the reader study are summarized on a breast basis. Multiple findings could be recorded for a single breast; therefore, totals are not shown.

Mammography findings	Cancer	Not cancer
Mass, n	7	3
Asymmetry, n	7	0
Distortion, n	11	0
Calcifications, n	10	0

It is important to note that patients in the malignant and benign groups used digital mammography images from the most recent examination date before surgery or biopsy, including those taken at other facilities. The distribution of equipment across vendors is summarized in Table 7.

**Table 7:** Mammography systems used in the study Mammography system information was obtained from Digital Imaging and Communications in Medicine (DICOM) metadata recorded at the time of image acquisition.

Corporate name	Product name	number
Canon Medical Systems	MAMMOREX Pe^.^ru^.^ru DIGITAL	7
Carestream Health	unknown	1
FUJIFILM	unknown	20
GE HealthCare	Senographe Essential	216
GE HealthCare (Japan)	Senographe Pristina	2
GE HealthCare (Japan)	Senographe DS	15
Hologic	Lorad Selenia	3
Hologic Japan	Selenia Dimensions	22
KONICA MINOLTA	unknown	2
Siemens	Mammomat Inspiration	5
unknown	unknown	45

Validation of the AI-CAD

Validation of the dataset was performed by two radiologists with 11 and two years of clinical experience. First, they jointly reviewed each mammography image together with clinical information - including pathology specimen characteristics, lesion location, and preoperative MRI findings - to determine the GS diagnosis; consensus was reached through discussion. Each case was then assigned a final BI-RADS category, which served as the reference standard (GS) for comparison with AI-CAD output. For cases in which no radiographic abnormality was identifiable even upon detailed human inspection, and where clinical or pathological information suggested that the lesion may not have manifested on mammography, we assigned BI-RADS category 1 in the GS to reflect true imaging negativity. This distinction was made to avoid misrepresenting these inherently non-visible lesions as false negatives in AI evaluation.

Next, they compared the GS with the results of the AI-CAD to verify whether the regions showing significant abnormality scores in the AI-CAD matched the lesions indicated by the GS. Three diagnostic radiologists with 11, six, and six years of experience independently assessed and assigned BI-RADS background breast density categories for each case.

Reporting standards

This study was conducted in accordance with the STARD 2015 and the CLAIM 2024 update for reporting diagnostic accuracy studies for AI in medical imaging. We also considered relevant items from the in-development STARD-AI initiative where applicable, but as STARD-AI remains under development, full adherence is not yet possible.

Radiological assessment

A total of 13 readers participated in the study, including seven radiologists (three radiologists specializing in diagnostic radiology with 21, 16, and eight years of experience; two radiologists specializing in radiology with six years of experience each; and two radiology residents with four and three years of experience) and six breast surgeons (with 23, 18, 11, three, three, and two years of experience) with varying levels of experience. Each participant independently read the same 40 randomly selected cases (21 malignant, nine benign, and 10 normal). Readers were blinded to the reference standard. For each case, they performed two consecutive reading steps.

Step 1 (AI-Unaided Reading)

Readers independently reviewed each mammography without the AI-CAD output. They assigned the findings, calculated using the 11-point likelihood of malignancy (LOM) scores, where 0 indicated no suspicion of malignancy and 10 indicated definite malignancy, and selected a BI-RADS category separately for the left and right breast of each case.

Step 2 (AI-Aided Reading)

Immediately afterward, readers reviewed the AI-CAD heatmaps and abnormality scores, then re-assigned an LOM score and BI-RADS category for the same case.

All 13 readers interpreted the same 40 cases in a sequential design. Each case was first read unaided, followed immediately by AI-aided interpretation after reviewing the AI heatmap and abnormality score. Readers were blinded to clinical outcomes and the consensus gold standard during unaided reads. The 40 cases were randomly sampled from the validation pool but intentionally enriched (21 malignant, nine benign, 10 normal) to stabilize receiver operating characteristic curve (ROC) estimation while keeping the reading workload manageable. No washout period and no formal AI familiarization session were implemented. All scoring systems used for reader performance evaluation in this study are publicly available and do not require additional licensing for academic use.

Statistical analysis

For mammography evaluations by radiologists, the BI-RADS category scores of 1 and 2 were classified as “negative” and 3, 4, and 5 were “positive.” Based on prior research, the abnormality score cutoff of the AI-CAD was set at 10, which classifies scores <10 as “negative” and scores ≥10 as “positive.” Sensitivity and specificity were calculated based on true-positive classifications defined by pathological diagnosis for each breast. Diagnostic performance (AI-unaided vs. AI-aided) was evaluated using an MRMC nonparametric U-statistic approach. For each condition, 95% confidence intervals (CIs) were calculated using Wilson intervals for sensitivity and specificity, and the Hanley-McNeil method for the area under the receiver operating characteristic curve (AUC). In the reader study, average AUCs and paired differences (AI-aided minus unaided) were computed with 95% CIs, and comparisons across readers used the Wilcoxon signed-rank test. Per-reader AUC ranges and interquartile ranges (IQRs) were also summarized.

Statistical analyses were performed using iMRMC (R implementation for multi-reader ROC analysis) and EZR version 1.62 (an R Commander extension) [20,21].

## Results

AI-CAD diagnostic accuracy

Patient characteristics are presented in Tables 1-3. The study included 338 patients with 676 breasts as the target data for validation. Background breast density was broadly distributed, with most malignant cases presenting as heterogeneously dense. Among malignant lesions, invasive ductal carcinoma was the most common histology. Imaging features included masses, asymmetry, architectural distortion, and suspicious calcifications, with architectural distortion being notably frequent in cancers.

In the validation cohort of 676 breasts (127 cancers, 549 non-cancers), the AI achieved an AUC of 0.897 (95% CI 0.860-0.934), sensitivity of 0.787 (100/127; 95% CI 0.708-0.850), and specificity of 0.898 (493/549; 95% CI 0.870-0.921). When excluding 10 cancers classified as “non-visible,” the AUC increased to 0.930 (95% CI 0.897-0.963). These results show that AI-CAD achieved diagnostic performance comparable to that of expert radiologists.

Radiological assessment results

The characteristics of the patient groups used for reading are presented in Tables 4-6. Forty representative cases (21 malignant, nine benign, 10 normal) were selected for the reader study. Malignant cases included 15 invasive ductal carcinomas and seven ductal carcinoma in situ (DCIS), with dense breast tissue predominating. Reader performance with and without AI assistance is summarized in Table 8.

**Table 8:** Reader performance with and without AI assistance Mean area under the receiver operating characteristic curve (AUC) values are shown for unaided and AI-aided readings. (A) Results based on Breast Imaging Reporting and Data System (BI-RADS) category scores [10]. (B) Results based on likelihood of malignancy scores. Comparisons of AUC values were performed using the Wilcoxon signed-rank test. Z statistics and corresponding p-values are presented.

	Unaided mean AUC (95% CI)	AI-aided mean AUC (95% CI)	ΔAUC (95% CI)	p-value	Per‑reader AUC range (unaided)／(AI‑aided)
A	0.750 (0.735–0.765)	0.756 (0.732–0.778)	+0.006 (−0.009–0.021)	0.505	0.708–0.797 / 0.708–0.802
B	0.750 (0.735–0.768)	0.761 (0.739–0.773)	+0.011 (−0.001–0.023)	0.110	0.711–0.803 / 0.727–0.813

Across 13 readers and 40 enriched cases, the mean AUC based on BI-RADS categories increased from 0.750 (range 0.708-0.797; IQR 0.735-0.765) to 0.756 (range 0.708-0.802; IQR 0.732-0.778), with a mean difference of +0.006 (95% CI −0.009 to +0.021; p=0.505). Using LOM scores, mean AUC increased from 0.750 (range 0.711-0.803; IQR 0.735-0.768) to 0.761 (range 0.727-0.813; IQR 0.739-0.773), a difference of +0.011 (95% CI −0.001 to +0.023; p=0.110). These small improvements did not reach statistical significance.

**Figure 2:**
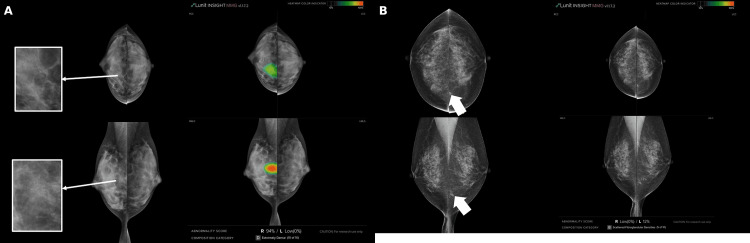
Representative cases demonstrating the impact of AI assistance (A) A case in which artificial intelligence (AI) assistance increased reader confidence by highlighting subtle calcifications in dense breast tissue. (B) A case in which AI assistance led to underestimation of malignancy due to a low abnormality score and unidirectional indication, illustrating a potential limitation of AI-based computer-aided detection.

Figure 2 illustrates examples where AI support influenced reader decisions.

In one case, AI assistance improved reader confidence in detecting subtle calcifications (Figure 2A), while in another, it led to underestimation of a malignant lesion in several readers (Figure 2B), highlighting both the benefits and limitations of AI-CAD in clinical interpretation.

## Discussion

Analysis of the validation data indicated that, even with AI-CAD assistance, lesions with subtle mammographic features remain difficult to detect. When such cases were excluded, the performance of the AI-CAD was comparable to that of the radiologists in terms of detection capability. Specifically, when cases in which radiologists visually judged lesions as challenging to identify were excluded, the diagnostic performance of the AI-CAD showed a sensitivity of 84% and a specificity of 89%. The diagnostic accuracy observed in this study fell within the range reported for the same AI system in prior investigations, which described a sensitivity of 82% and a specificity of 90%, even though different software versions were used [22].

Notably, while previous research primarily examined non-Japanese populations, our study specifically evaluated the system’s performance in a Japanese cohort, where 65% of participants presented with high-density breast tissue (heterogeneous or extremely dense)-a characteristic that typically poses greater challenges for mammographic interpretation. Qualitative case examples suggest potential effects of AI on reader confidence in dense breasts, although this was not formally measured.

Direct statistical comparisons with prior studies are limited by differences in sample size and case composition; however, the observation of comparable diagnostic performance within a Japanese cohort remains clinically noteworthy, suggesting that the AI-CAD maintains its effectiveness across diverse population groups. These findings suggest that the diagnostic support capabilities of the AI-CAD may be robust across diverse populations. Further investigation into its potential for broader clinical implementation across different demographic contexts may, therefore, be warranted.

In the radiological assessment protocol, the MRMC study using 40 Japanese diagnostic cases, AI assistance did not degrade reader performance, and small AUC improvements (+0.006 to +0.011) did not reach statistical significance. Given the limited case number, the study was underpowered to detect such small effect sizes, and type II error cannot be excluded. The enriched case composition and absence of a washout period may introduce spectrum and design bias. Our dataset, collected over five years ago, may not fully represent current Japanese screening practice. Thus, the findings should be interpreted as an initial external validation suggesting that AI trained outside Japan can maintain accuracy in Japanese patients. We refrain from generalizing to national screening programs. Prospective, adequately powered screening studies incorporating workflow, reader time, and cost analyses are needed to determine clinical impact.

Notably, in cases with calcifications within the lesions, particularly in dense breasts where such features were prone to being overlooked (Figure 2A), AI-CAD support helped the readers to detect these findings more reliably and increased their diagnostic confidence in identifying malignancy.

Conversely, there are points of reflection in the case shown in Figure 2B. While readers could identify findings in both the craniocaudal and mediolateral oblique (MLO) views, their confidence in malignancy appeared low. In contrast, the AI only indicated findings in the MLO view and provided a low abnormality score of 12. Category reassignment was observed among several readers, with changes occurring both from lower to higher and from higher to lower categories. This may be due to their interpretation of the image as part of the normal structures, possibly due to low abnormality scores and unidirectional indications. Nevertheless, considering the actual findings, the AI-CAD should not have downgraded this case to Category 1, as this would have been considered a positive judgment.

Cases in which radiologists are uncertain based on individual findings may lead to differing judgments based on their understanding and trust in the AI-CAD. To achieve results consistent with the AI-CAD, emphasizing the interpretation of abnormal scores and providing training for such judgments may be necessary.

Limitations

The present case-control design has inherent limitations when applied to the evaluation of AI systems intended for screening purposes. In this study, a significant proportion of malignant and benign cases was evaluated based on pathological results, and there were many cases in which assessment based solely on images was challenging. These design choices are now acknowledged as potential sources of bias.

To conduct the reading experiment efficiently, we intentionally limited the sample size to 40 cases, considering practical constraints such as clinical workflow and the potential for reader fatigue. While the relatively small number of cases may have introduced some variability in the results, this design choice offered a pragmatic balance, allowing participants to complete the session in a single sitting during routine clinical hours without compromising diagnostic consistency. Given the small sample size (40 cases, 13 readers), this study had limited statistical power to detect small AUC differences. Prior MRMC studies suggest that at least 100 cases would be needed to reliably detect AUC differences of 0.01 or less with adequate power (>0.80) [23]. Therefore, our study should be interpreted as a feasibility assessment rather than a definitive evaluation of AI benefit.

A dedicated practice period for familiarization with the AI-CAD was not allocated prior to the reading sessions. The variability in participants’ prior experience with AI-CAD, in conjunction with the aforementioned practical constraints, rendered the identification of consistent patterns in diagnostic accuracy improvement a challenging endeavor. Feedback from the participants indicated that the AI-CAD increased confidence in diagnosing malignancy in cases suspected to be malignant or decreased confidence in diagnosing non-malignant lesions. Reading time and changes in diagnostic confidence were not quantitatively assessed in this study; therefore, potential time-saving effects could not be evaluated.

Further research, including larger sample sizes, long-term studies, and comparisons of reading times in real-world reading environments, is warranted to facilitate clinical implementation of AI-CAD for mammography screening in Japan.

## Conclusions

This study demonstrated that an AI-CAD system trained on non-Japanese data maintained diagnostic performance when applied to mammography images from Japanese women. Although AI assistance did not yield statistically significant improvements in reader performance within this limited reader study, the results suggest the feasibility of applying externally trained AI-CAD systems to Japanese clinical settings. Larger, prospective screening studies are warranted to clarify their clinical impact.
